# Leptin in Osteoarthritis and Rheumatoid Arthritis: Player or Bystander?

**DOI:** 10.3390/ijms23052859

**Published:** 2022-03-05

**Authors:** Djedjiga Ait Eldjoudi, Alfonso Cordero Barreal, María Gonzalez-Rodríguez, Clara Ruiz-Fernández, Yousof Farrag, Mariam Farrag, Francisca Lago, Maurizio Capuozzo, Miguel Angel Gonzalez-Gay, Antonio Mera Varela, Jesús Pino, Oreste Gualillo

**Affiliations:** 1 SERGAS (Servizo Galego de Saude) and IDIS (Instituto de Investigación Sanitaria de Santiago), NEIRID Lab (Neuroendocrine Interactions in Rheumatology and Inflammatory Diseases), Research Laboratory 9, Santiago University Clinical Hospital, 15706 Santiago de Compostela, Spain; djidji.aiteldjoudi@gmail.com (D.A.E.); sitoalcorba@gmail.com (A.C.B.); maria.gonzalez3112@gmail.com (M.G.-R.); clararf94@gmail.com (C.R.-F.); yousof.farrag@gmail.com (Y.F.); mariam.r.farrag@gmail.com (M.F.); jesus.pino.minguez@sergas.es (J.P.); 2 International PhD School, University of Santiago de Compostela (EDIUS), 15706 Santiago de Compostela, Spain; 3 Molecular and Cellular Cardiology Group, SERGAS (Servizo Galego de Saude) and IDIS (Instituto de Investigación Sanitaria de Santiago), Research Laboratory 7, Santiago University Clinical Hospital, 15706 Santiago de Compostela, Spain; francisca.lago.paz@sergas.es; 4 National Health Service, Local Health Authority ASL 3 Napoli Sud, Department of Pharmacy, Ercolano, 80056 Naples, Italy; m.capuozzo@aslnapoli3sud.it; 5 Hospital Universitario Marqués de Valdecilla, Epidemiology, Genetics and Atherosclerosis Research Group on Systemic Inflammatory Diseases, IDIVAL, University of Cantabria, Avenida de Valdecilla s/n, 39011 Santander, Spain; miguelaggay@hotmail.com; 6 SERGAS, Servizo Galego de Saude, Santiago University Clinical Hospital, Division of Rheumatology, 15706 Santiago de Compostela, Spain; antonio.mera.varela@sergas.es

**Keywords:** leptin, leptin receptor, rheumatoid arthritis, osteoarthritis, inflammation

## Abstract

White adipose tissue (WAT) is a specialized tissue whose main function is lipid synthesis and triglyceride storage. It is now considered as an active organ secreting a plethora of hormones and cytokines namely adipokines. Discovered in 1994, leptin has emerged as a key molecule with pleiotropic functions. It is primarily recognized for its role in regulating energy homeostasis and food intake. Currently, further evidence suggests its potent role in reproduction, glucose metabolism, hematopoiesis, and interaction with the immune system. It is implicated in both innate and adaptive immunity, and it is reported to contribute, with other adipokines, in the cross-talking networks involved in the pathogenesis of chronic inflammation and immune-related diseases of the musculo-skeletal system such as osteoarthritis (OA) and rheumatoid arthritis (RA). In this review, we summarize the most recent findings concerning the involvement of leptin in immunity and inflammatory responses in OA and RA.

## 1. Introduction

Rheumatic disorders include many different forms of arthritis, such as osteoarthritis (OA) and rheumatoid arthritis (RA), that lead to pain, swollen joints, and progressive damage to articular cartilage [1].

OA is a degenerative inflammatory joint disorder impacting mainly older populations with major socioeconomic impacts for health and social-care systems. It is considered a whole-joint disease with alterations in articular cartilage, subchondral bone, ligaments, and synovium [2]. Its prevalence rises with age and is related to sex (women are likely more exposed than men) and it affects small (hand) and large (knee and hip) joints [3]. RA is a severe chronic and progressive autoimmune disorder characterized by synovium inflammation. It affects diarthrodial joints such as the hand, knee, and hip, resulting in systemic complications, joint and bone destruction, and progressive disability [4]. Although the pathological mechanisms involved in OA and RA are different, the onset and progression of both diseases are associated with inflammation, immune mechanisms, and metabolic factors [5,6]. Obesity, a major health problem, has been described as a risk factor in RA and OA development [7,8]. In addition, mechanical loading and inflammatory mediators such as adipose-tissue-derived cytokines (adipokines) have been reported as a link between obesity and OA [9]. Adipokines including leptin, adiponectin, visfatin, and resistin are cytokine-like-hormones secreted principally by white adipose tissue (WAT) [10]. Through their endocrine, autocrine, or paracrine actions, they are implicated in several physiological and pathological processes and lead to a “low grade inflammatory state” in overweight subjects [11]. Indeed, they are demonstrated to be involved in the pathogenesis of rheumatic diseases by the modulation of the inflammatory process in the joint, the imbalance between catabolic and anabolic factors, and the remodeling of bone and cartilage [1].

Adipokine levels have been reported to be greatly increased in serum and synovial fluid (SF) of RA patients [12,13]. Furthermore, several studies outlined the implication of adipokines in the progression and severity of OA and the chronic inflammation in articular joints [14,15].

Leptin is the main adipokine secreted by adipose cells. It exerts its role by binding to the long isoform receptor Ob-Rb and transducing the signa through the Janus kinase/signal transducer and activator of transcription (JAK/STAT) signaling pathway [16]. In addition to its evident role in regulating energy homeostasis and food intake, it also has pleiotropic functions [17]. Leptin is implicated in both adaptive and innate immunity. Increasing evidence suggests that leptin exerts potent modulatory actions in the network of factors implicated in the pathophysiology of rheumatic diseases such as OA and RA [18]. This review recapitulates the most relevant data regarding the involvement of leptin in these two diseases.

## 2. Leptin Physiology

Leptin is a 16 Kda protein discovered in 1994 by Friedman and collaborators by positional cloning in the *ob/ob* mouse gene, a homologue of the *LEP* human gene, which encodes a protein of 167 amino acids [16]. It belongs to the class I helical cytokine family which includes growth hormone (GH), leukemia-inhibiting factor (LIF), granulocyte colony stimulating factor (G-CSF), interleukins (IL) 2, 3, 4, 5, and 10, and comprises four α antiparallel helices A, B, C and D [19]. Leptin is predominantly synthetized by WAT, however, other sites such as skeletal muscle, stomach, bone marrow, brain, mammary epithelial cells, placenta, ovary, and cartilage have also been reported to produce detectable amounts of leptin [20,21,22]. Leptin circulating concentrations depend on nutritional status and follow a circadian rhythm and show low levels from morning to afternoon and high amounts between midnight and early morning [23]. Many factors are implicated in the regulation of its synthesis and secretion. Glucocorticoids have been shown to be one of the main stimulators of leptin secretion by acting directly on the white adipose tissue [24]. Insulin has also been reported to elevate mRNA expression of leptin in human and rat adipocytes. Moreover, hyperinsulinemia in humans is associated with an increase in leptin amounts exerting long-term effects [25]. Cytokines, toxins, and sex steroids also regulate leptin secretion. Leptin levels are higher in females than in males of the same age and BMI, due in part to the modulatory actions of estrogens and androgens, respectively [26,27]. Furthermore, prostaglandins, via the cyclooxygenase 2 (COX2) pathway, may be involved in leptin secretion. As matter of fact, NS-398, an inhibitor of COX2, blocking the arachidonic acid conversion to prostaglandins E2 (PGE2), is able to down-regulate leptin secretion [28,29]. Catecholamine release and β-adrenergic receptor activation, the elevation in cyclic AMP induced by adenylate cyclase activators, melatonin, and valproic acid are involved in the down-regulation of leptin secretion [19,30].

Since its discovery, leptin has been recognized as a single hormone regulating body weight and energy balance, yet recent studies have proved its implication in multiple biological functions such as glucose metabolism, hematopoiesis, reproduction, and interaction with the immune system and consequently, it has been recognized as a pleiotropic factor [26]. Leptin regulates food intake and energy homeostasis; elevation in its levels leads to the inhibition of hypothalamic orexigenic peptides (agouti-related peptide (AgRP), and neuropeptide Y (NPY)), and the activation of anorexigenic peptides (cocaine and amphetamine-related transcript (CART) and proopiomelanocortin (POMC). It regulates lipid metabolism by enhancing lipolysis and inhibiting lipogenesis [31]. Leptin is implicated in both innate and adaptive immune responses. It promotes the synthesis and secretion of pro-inflammatory cytokine IL-6, tumor necrosis factor (TNFα), and IL-12, phagocytosis activity in macrophages, and natural killer (NK) cell proliferation. It also prevents neutrophil apoptosis through phosphatidylinositol 3-kinase (PI3K) and mitogen-activated protein kinase (MAPK) pathways. Furthermore, it enhances T-cell proliferation and memory-T-cells differentiation to T-helper (Th1), inhibits regulatory-T-cell (Treg) proliferation, and activates the mammalian target of rapamycin (mTOR) pathway [32,33]. It is also associated, with other adipokines, with several autoimmune diseases such as type1 diabetes, inflammatory bowel disease, systemic lupus erythematosus (SLE), and Crohn’s disease [34]. In its reproductive functions, leptin has been reported to regulate the secretion of gonadotropin hormones and follicular and luteal steroidogenesis [35]. In homozygous female *ob/ob* mice, administration of leptin suppressed the reproductive defect and corrected ovulation, pregnancy, and parturition [36].

## 3. Leptin Receptors

Leptin receptor (LepR) was identified by cloning from mouse choroid plexus [37]. It belongs to the class I cytokine receptor superfamily which includes IL-6, leukemia-inhibiting factor, and granulocyte-colony-stimulating factor. Six isoforms of LepR (four short, one long, and one soluble) have been identified, resulting from alternate splicing during *db* gene expression. Leptin receptors are expressed ubiquitously with a distinct repetition between the short and long isoforms. Ob-Ra is expressed in almost all the tissues while Ob-Rb is mainly expressed in the hypothalamic nuclei, but it is also expressed at lower levels in peripheral tissues such as liver, pancreas, lungs, heart kidneys, adipocytes and immune cells [19]. These isoforms contain the same amino-terminal ligand-binding domain and different carboxyl terminal regions. Ob-Rb, the longest isoform, contains specific intracellular domains known as box1, box2, and box3 regions and it is the only isoform able to induce a full signal transduction. Box 1 contains proline residues that are essential for the recruitment of JAK2. Box 3 has a role in the recruitment and activation of STAT3 [31]. In addition to Box regions, it contains four tyrosine residues (Tyr ^974^, Tyr ^985^, Tyr ^1077^, and Tyr ^1138^). The phosphorylation of these tyrosines allows the binding of proteins containing a SH2 (SRC-like homology 2) domain, such as STAT, SHP-2 (SH2-domain-containing protein tyrosine phosphatase) and SOCS (suppressors of cytokine signaling) [38]. The Ob-Rb intracellular domain consists of 303 amino acids and shares the same sequence of the first 29 amino acids with the short isoforms containing from 30 to 40 amino acids [37]. Indeed, the other two cytoplasmic tyrosines of murine Ob-Rb, Y985 and Y1077, have been shown to bind other intracellular signaling molecules [39]. The extracellular ligand-binding domain is identical for the long and short isoforms of lepR. It consists of 816 amino acids and contains seven structural domains. The first two domains (1 and 2) adopt fibronectin type III (FNIII) folds and form the cytokine recognition module CRH1 (cytokine receptor homology1). Domain 3 encompasses the immunoglobulin-like fold (IGD). Domains 4 and 5 constitute the second cytokine recognition module (CRH2) that acts as a binding site for leptin. The two last domains (6 and 7) have also FNIII folds [40]. Ob-Re, the smallest isoform possesses only an extracellular domain and it is considered a soluble binding protein [41].

### Leptin Receptor Signaling

Receptor activation is initiated by leptin binding and then activating multiple signaling pathways. As LepR is not dotted with intrinsic tyrosine kinase activity, it enrolls a cytoplasmic Janus kinase JAK2 [16]. Leptin binding activates JAK2 and leads to its autophosphorylation. Activated JAK2 phosphorylates three tyrosine residues Tyr ^985^, Tyr ^1077^, and Tyr ^1185^ providing docking sites for signaling molecules. The JAK signal transducer and activator of transcription (JAK/STAT) pathway involves STAT recruitment to the phosphorylated tyrosine residues. Activated STAT then forms dimers and is translocated to the nucleus where it enhances gene transcription [42]. Other signaling pathways, such as PI3K and MAPK pathways, are also involved in leptin signaling. The MAPK pathway is activated by the binding of the phosphorylated residue Tyr ^985^ to the SH2 domain of protein tyrosine phosphatase (SHP2), while the PI3K pathway is activated by the phosphorylation of the insulin receptor substrate (IRS) [43].

## 4. Leptin and Osteoarthritis

OA pathogenesis is associated with local and systemic factors including age, sex, genetics, obesity, joint injury, and mechanical stress. The association of these multiple factors leads to a degeneration of articular joints complicated by inflammatory reactions [42]. In the early phases of the disease, the composition of the cartilage matrix dramatically changes, which enhances its disruption susceptibility. The first alteration is the fibrillation of the superficial cartilage layer leading to fissures in deep layers that may be extended to subchondral bone and alteration of the architecture of the cartilage. In addition, a change in the morphology of chondrocytes is also involved in this process, where they become larger [43]. The release of cartilage fragments and the hypertrophy of chondrocytes in an attempt to repair, enhance chondrocyte synthetic activity and provoke the release of pro-inflammatory mediators by the adjacent synovium, initiating the inflammatory response. Proteoglycan breakdown is enhanced and activated, and synoviocytes and articular cartilage up-regulate metalloproteinase (MMP) gene expression leading to an imbalance between metalloproteinase production and tissue inhibitor of metalloproteinases (TIMP) [43,44]. Cytokines, first produced by the synovial membrane, support the inflammatory process and the global catabolic effect. The release of cytokines to cartilage via SF leads to chondrocyte activation and production of pro-inflammatory mediators. IL-1, IL-6, TNFα, and IL-17 are the main cytokines involved in OA pathogenesis. NOS2 and COX2 are also involved [45]. OA is also accompanied by bone damage and synovium inflammation. Alterations in osteoblast and osteoclast activity lead to an imbalance of bone turnover [46]. Lymphocyte infiltration to the synovium, angiogenesis, and a hyperplastic synovial lining layer are indicative of synovitis [44].

### 4.1. Leptin in Serum and SF of OA Patients

Diverse clinical studies have demonstrated the implication of serum/plasma and/or SF leptin levels in OA (Table 1). Min et al. showed an enhanced expression of leptin in knee OA patients compared to control in both genders with higher expression in female patients compared to male [45]. In articular cartilage isolated from OA patients, leptin levels were more elevated in SF than in serum and both exhibited a positive correlation with BMI [46]. Furthermore, leptin showed high SF and serum levels in both knee and hip OA and correlated with SF concentrations of IL-6 [47]. Ob-Rb leptin receptor expression was enhanced in OA chondrocytes compared to normal chondrocytes while Ob-Ra mRNA was expressed equally in both OA and healthy cartilage [46].

Leptin and its receptor are associated with stage of OA disease and related pain. Notably, high leptin concentrations in SF of OA patients are correlated with joint pain [48]. mRNA expression of leptin and its receptor Ob-Rb was higher in OA cartilage compared to both mildly affected and no OA cartilage suggesting that Ob-Rb is expressed by highly damaged cartilage [46]. Another study by Ku et al. in SF of patients with different stages of OA showed the highest concentration in the most advanced stage of the disease [49]. Moreover, leptin levels correlated with WOMAC-pain score and radiographic stage of knee OA where high levels were observed in more advanced stages of the disease. This indicates that leptin promotes OA progression [50].

Leptin is associated with OA structural progression. Notably, its expression levels are correlated with the degree of cartilage destruction and observed in the fibrillation and areas of matrix depletion [51]. Furthermore, leptin levels are correlated with elevated cartilage volume waste in the lateral and medial compartments. Patients with higher baseline leptin levels are more likely to be susceptible to total knee replacement suggesting a role of leptin as a biomarker of OA and in disease prognosis [52]. Moreover, a cross-sectional and longitudinal study by Stannus et al. revealed that leptin levels consistently correlated with cartilage thickness reduction. The adiposity and cartilage thickness association was decreased in all sites after adjustment for leptin suggesting a mediating role for leptin and a key contribution to cartilage loss [53].

In hand OA, the association of leptin levels with the disease is controversial. In the study by Yusuf et al., it has been reported that serum leptin levels were not linked to the progression of OA, whereas adiponectin reduced the risk of hand OA progression by 70% [54]. Similar results were reported in the HANES III cross-sectional study in 2477 patients where no significant difference was recorded in serum leptin levels between asymptomatic, symptomatic, and no hand OA in both genders [55]. By contrast, Morales Abaunza et al. reported a significant elevation of serum leptin levels in hand OA patients in comparison with healthy controls [56].

**Table 1:** Summary of clinical studies on leptin levels in serum and SF of OA patients.

Author	Sample	Patients	Leptin Levels	Relation with the Disease
Dumond et al. (2003) [51]	Synovial fluid	20 OA, 2H	Detected in SF and correlated with BMI	Yes
Simopoulou et al. (2007) [46]	SF/Serum	17 OA, 5H	Significantly much higher in OA patients	Yes
Ku et al. (2009) [49]	SF	42 OA, 10H	Significantly higher in OA patients	Yes
Min et al. (2020) [45]	Serum	148 OA, 101H	Significantly higher in OA patients	Yes
Lübekke et al. (2013) [48]	SF from hip and knee	219	High leptin levels in SF were correlated with joint pain	Yes
Xiong et al. (2018) [57]	SF	13 OA, 7H	Significantly higher in OA patients than all the other groups	Yes
Kroon et al. (2019) [58]	Serum from hand and knee	6408	Leptin levels were positively associated with OA	Yes
Massengale et al. (2012) [55]	Serum from hand	2477	No significant difference between symptomatic, asymptomatic, and no hand OA	No
Yusuf et al. (2011) [54]	Serum from hand	248	Not associated with hand OA progression	No
De Boer et al. (2012) [14]	Serum from knee	172 OA, 132H	Significant difference between OA patients and control	Yes
Morales Abaunza et al. (2020) [56]	Serum from hand	44 OA, 30H	Significantly higher in patients with hand OA	Yes
Bas et al. (2014) [47]	Serum and SF from hip and knee	112 hip OA, 92 knee OA	Higher in knee OA than in hip OA joints	Yes

SF, synovial fluid; OA, osteoarthritis; H, healthy controls.

### 4.2. Leptin in Metabolism and Inflammation of Articular Cartilage

**Figure 1:**
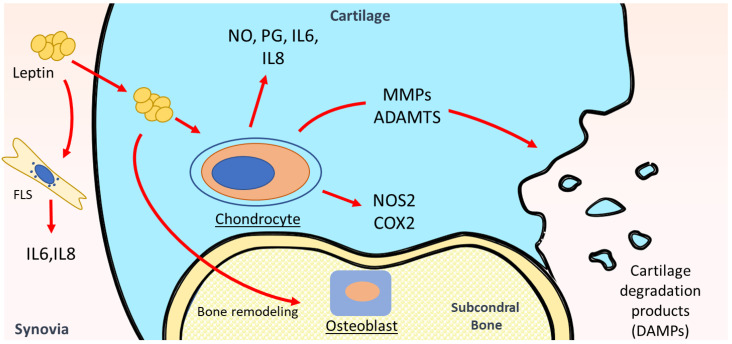
Leptin impact in OA pathophysiology. Leptin modulates the inflammatory environment in the joint, the imbalance between catabolic and anabolic factors, and remodeling of bone and cartilage. It also activates chondrocytes and production of pro-inflammatory mediators including IL-6, IL-8, NO, PG, NOS2, and COX2 and up-regulates metalloproteinases (MMP) production leading to extracellular matrix (ECM) degradation. It modulates cytokine production in synovial fibroblasts and bone remodeling imbalance exacerbating cartilage destruction and OA progression.

Leptin was reported to be a key regulator in cartilage metabolism [59] (Figure 1). Progressive articular cartilage destruction is a main characteristic of OA. It results from an imbalance between anabolic and catabolic factors in joints leading to degradation of the extracellular matrix (ECM) by enhanced levels of matrix-degrading enzymes [2]. Recent studies showed the involvement of leptin as a key mediator in the pathophysiology of OA via up-regulation of MMP production in OA joints. Leptin alone or combined with pro-inflammatory cytokines (IL- or TNFα) enhanced collagen degradation. The collagen release was followed by high regulation of collagenolytic MMP-9 and gelatinolytic MMP-2 activities. The same group showed elevated mRNA expression of the collagenases MMP-1 and MMP-13 when leptin was added to human primary chondrocytes. A synergetic effect was noted in the combination of leptin and IL-1 resulting in the activation of STAT1, STAT3, STAT5, MAPK (JNK, Erk, and p38), Akt, and NF-κB signaling pathways [60]. Similar results were observed when leptin was combined with IL-1β, leading to the enhancement of MMP1, 3, and 13 levels in OA cartilage. Furthermore, leptin levels were positively associated with MMP-1 and MMP-3 levels in SF from OA patients [61]. Multiple intracellular signaling pathways were involved in leptin regulation of MMPs. NFκB, JNK, and PKC regulated leptin-induced production of MMP-1, MMP-3, and MMP-13 in OA cartilage, JAK3 was involved in MMP-3 and MMP-13, while p38 was involved in MMP-1 and MMP-13 production [61].

In addition to the matrix-degrading enzymes, catabolic cell behavior is also associated with enhanced pro-inflammatory cytokines such as IL-1β and TNFα, which are reported to have a pivotal role in OA pathogenesis. Leptin induced dose-dependent IL-1β production after 7 days of culture, as well as MMP-9 and MMP-13 protein expression in a dose-dependent way in osteoarthritic and normal human chondrocytes [46].

Nitric oxide (NO) is involved in different cartilage functions, it increases modulation of MMPs, apoptosis, and dedifferentiation of chondrocytes. Leptin increased NO, PGE2, IL-6 and IL-8 production, and nitric oxide synthase 2 (NOS2) and COX2 protein expression in human OA cartilage. NO inhibition led to a decrease in leptin-induced production of these inflammatory mediators while exogenous addition of NO reversed this effect suggesting that the leptin effect is dependent on NO. NFκB, PKC, JAK3, and JNK inhibited leptin-induced NO, IL-6, and IL-8 production, while PGE2 production was inhibited by suppressors of NFκB, JNK, and MAPKs (p38 and ERK1/2) [62]. Another study by Otero et al. revealed that leptin synergistically, in combination with INF-γ, induced NO accumulation and NOS2 expression in human OA chondrocytes and the murine ATDC5 chondrogenic cell line via the JAK2 signaling pathway [63]. The same authors reported, in another study, an activation of NO production through an up-regulation of NOS2 at both mRNA and protein expression levels in human primary chondrocytes and mature and hypertrophic ATDC5 chondrocytes after a co-stimulation with leptin and IL-1, one of the most relevant cytokines involved in pathogenesis of OA [64]. The JAK2 pathway was involved in the early steps of NOS2 expression resulting in enhanced NO production in primary human and ATDC5 chondrocytes by the synergistic effect of leptin and INFγ [63] or leptin and IL-1 [65]. Furthermore, leptin intraarticular injection into rat knee joint up-regulated both mRNA and protein expression of IGF1 and TGFβ1 and enhanced proteoglycan production in rat cartilage in a dose-dependent manner [51].

The long form of the leptin receptor Ob-Rb is highly expressed in OA cartilage. Its overexpression may contribute to cartilage degradation by activating the leptin pathway. Treatment of Ob-Rb-overexpressing chondrocytes by physiological doses of leptin inhibited cell proliferation and led to chondrocytes senescence via activation of P53/P21 pathway. Furthermore, leptin inhibited autophagy and activated the mTOR pathway in chondrocytes. Blockade of mTOR restored autophagy and partially reversed chondrocyte senescence [66]. These results indicate that leptin enhances chondrocyte senescence through activating the mTOR pathway in OA cartilage.

### 4.3. Leptin in Other Intraarticular Tissues of OA

In addition to articular cartilage damage, the pathophysiology of OA also involves bone alterations and synovium inflammation. OA synovium activation leads to proteinase and cytokine release and exacerbates cartilage destruction [67]. Leptin promotes inflammatory cytokine production in OA synovial fibroblasts. It enhances IL-6 production through the binding to its receptor Ob-Rb and the activation of IRS-1, PI3K, AkT, and AP-1 signaling pathways [68]. IL-8 was also reported to increase in synovial fibroblasts by leptin through the same pathway [69], indicating that Ob-Rb/IRS-1/PI3K/AkT may be a common signaling pathway in leptin-induced gene expression in synovium. In another study, leptin did not directly affect IL-6 secretion in synovial fibroblast, however, it enhanced the cross-talk between chondrocytes and fibroblasts and then increased IL-6 secretion. This may explain the increased IL-6 amounts in the synovial fluid of OA subjects [70].

In a study of gene profiling in OA subchondral bone in humans, leptin was reported to be implicated in bone remodeling by osteoblasts (Ob) [71]. Leptin was found to be highly expressed in OA subchondral osteoblasts compared to normal Ob. In addition it enhanced cell proliferation and ERK 1/2 and p38 phosphorylation. The authors suggested that the increase of leptin production might be responsible for the abnormal phenotypic Ob observed in OA including increased alkaline phosphatase activity, osteocalcin release, and TGF-β1 levels [72]. Taken together, these results indicate that leptin is implicated in the bone remodeling imbalance occurring in OA.

Along with the synovium, the infrapatellar fat pad (IFP) has been shown to be the main source of adipokines in the joint, releasing high levels of leptin [73,74]. In a study of OA individuals with and without metabolic syndrome (MetS), Liu and colleagues reported that synovium and IFP secreted higher amounts of leptin in patients with MetS [75]. Gross et al. revealed that IFP enhanced the gene expression of pro-inflammatory mediators in both FLS and chondrocytes but not through leptin as its blocking did not change the cell response [76]. This suggests that IPF has a role in the inflammatory process of OA but the involvement of leptin in this deleterious effect is not well understood and further studies should be conducted to understand it.

### 4.4. Leptin and Obesity in OA

Obesity is a potent risk factor for OA incidence and progression [77]. Although it promotes the mechanical overload of weight-bearing joints, this is not the only link between obesity and OA. Accordingly, OA in the hand, a non-weight bearing joint, was demonstrated to increase in obese subjects in comparison to controls. Griffin et al. did not find any difference in OA incidence between leptin (*ob/ob*)- and leptin receptor (*db/db*)-deficient mice and control mice developing extreme obesity. These results imply that adiposity alone is not sufficient to generate knee OA and that leptin has a critical role in obesity-related OA [78]. Indeed, mRNA expression of leptin was significantly higher in OA cartilage of overweight patients than in normal weight ones and was positively correlated to BMI of the patients. Leptin levels showed a positive and significant correlation with BMI, and were more elevated in women than men in end-stage knee OA patients [14]. Leptin mediates the association of OA with obesity. Accordingly, leptin amounts were significantly higher only in knee OA obese patients (with BMI > 30) as compared with the control group. Obesity, in association with high levels of adipokines (leptin and resistin), was reported to promote earlier development of knee OA in younger patients [50].

In rats fed with a high fat diet (HFD), serum leptin levels increased in the early stage (week 5) preceding the increase in SF that was noted later (week 15) and the appearance of OA-like lesions in knees of HFD rats (week 27). MMP13 levels as well as the Mankin score were also remarkably increased over time. Furthermore, TLR4, associated with obesity-related inflammatory responses, is activated by leptin through the JAK2–STAT3-CD14 signaling pathway to promote OA. This suggests that increased serum levels are an initiating factor of obesity-related OA [79].

Taken together, these results indicate that obesity, characterized by excess adipose tissue, leads to elevated leptin production and generates the low-grade inflammation state that characterizes obesity, and then promoting metabolic disorders and autoimmune inflammatory diseases.

## 5. Leptin and Rheumatoid Arthritis

However, although predisposition to RA has been associated with genetic and environmental factors and epigenetic modifications [80], the initiation and the etiology of the disease is still elusive. Autoantibodies such as anticitrullinated protein antibody and rheumatoid factor (RF) are the first immune abnormalities detected, followed by joint damage starting in the synovial membrane. Synovium inflammation appears in the early stages of the disease after activation of endothelial cells that express adhesion molecules and chemokines following the infiltration of leukocytes through the synovium [81]. The clinical phase of RA starts with stimulation of the adaptive and innate immune systems. T and B cells and macrophages with the plethora of inflammatory cytokines mostly IL-6, TNFα, IL-17, and IL-1 play a potent role in the RA pathogenesis [82]. The synovial cells invasion, mostly by FLS that produce metalloproteinases, and the metabolic effects occurring in chondrocytes, lead to cartilage damage. Osteoclast activation by synovial cytokines, mostly the receptor activator of NF-κB ligand (RANKL), expressed in B cells, T cells, and fibroblasts, leads to an imbalance of bone turnover and bone erosion. These cellular and molecular events indicate clinical disease expression [83].

### 5.1. SF and Serum Leptin Levels in RA Patients

Leptin has been described to be implicated in RA pathogenesis. However, the results of clinical studies comparing serum or SF leptin concentrations in healthy individuals and RA patients are still ambiguous. Many authors have reported significant elevation of serum and SF leptin levels in RA patients compared to healthy controls [12,84,85,86,87], while others did not report difference in these levels (Table 2) [88,89,90,91]. Hizmetli and colleagues did not find any significant difference in SF and plasma leptin levels between the two groups of RA patients and healthy individuals and did not find a correlation to disease duration, ESR, CRP, RF, or erosive or non-erosive RA [88]. The same results were reported in other studies and no correlation was reported between leptin levels and inflammatory markers CRP, ESR, RF, leucocytes, and ANA [82,87]. Popa and collaborates reported, in addition, that plasma leptin concentrations were inversely correlated to inflammatory markers in RA patients suggesting that chronic inflammation in RA decreases leptin production. They also revealed that after a two weeks of treatment with anti-TNF, which is supposed to increase leptin concentrations, no difference was observed with baseline levels in either men or women despite IL6 and CRP diminution [90]. On the other hand, Tong et al., indicated that leptin treatment led to an increase in IL-8 and Ob-Rb mRNA expression in SF of RA patients [69]. Other studies also reported a significant correlation between serum leptin concentrations and reduced joint damage, DAS28, ESR, CRP, IL6, and TNFα levels, and RA duration [89,90,91,92,93]. Ambiguous results were also reported regarding the association of leptin concentrations with disease activity. Serum leptin levels were significantly higher in RA patients with high disease activity compared to those with lower activity and were associated with DAS28 and CRP levels [85,86,93,94,95]. In contrast, many studies reported a lack of correlation between leptin amounts and disease activity [84,87,91]. Abdalla et al. did not find any statistically significant difference between serum leptin levels in patients with low, moderate, and high disease activity. Similarly, Wislowska and collaborators showed no difference in serum leptin values in different stages of the disease and in high and low DAS28 [96]. Targońska-Stȩpniak showed that there was no association between leptin levels, disease duration, and DAS28, however, in the group of patients with long-standing RA (duration of more than 10 years), a positive correlation was reported between serum leptin levels, DAS28, and the number of tender joints, suggesting an association between elevated serum concentrations and the aggressive course of RA [97].

Concerning the association of leptin with disease duration, no correlation was reported between levels of leptin in RA patients and disease duration [87,89,90]. In contrast, Bokarewa and collaborators, showed a gradual increase in plasma and SF leptin levels with the increase of the disease duration [84].

Interestingly, SF leptin concentrations were more elevated in individuals with erosive RA than in those with non-erosive RA, while no significant difference was observed in plasma. The difference between plasma and SF levels was more pronounced in non-erosive RA patients [84]. Results confirmed by Olama and colleagues revealed that the SF/serum ratio was significantly higher in RA patients with radiologic erosions. The authors suggested that this decrease may be a local uptake, supporting the hypothesis of the potent protective role of leptin in the joint erosive process [92]. These discrepancies observed in the different studies may be related to different factors such as the confounding factors resulting from the co-existence of other autoimmune diseases in RA patients, methods of leptin measuring, small sample sizes, different treatments given, etc. [98].

**Table 2:** Summary of clinical studies on leptin levels in serum and SF of RA patients.

Author	Sample	Patients	Leptin Levels	Evidence of Relation with the Disease
Petra et al. (2020) [99]	Serum	84 RA, 44H	Significantly higher in RA patients than in controls	Yes
Lee et al. (2007) [86]	Serum	50 RA	RA patients had higher mean leptin levels	Yes
Bokarewa et al. (2003) [84]	Serum and SF	76 RA, 34H	Higher in RA patients	Yes
Rho et al. (2009) [93]	Serum	167 RA, 91H	Significantly higher in RA patients	Yes
Abdalla et al. (2014) [87]	Serum	60 RA, 30H	Significantly higher in RA patients	Yes
Olama et al. (2012) [92]	Serum and SF	40 RA, 30H	Increased in RA patients	Yes
Seven et al. (2009) [85]	SF and serum	20 RA, 25H	Significantly higher in RA patients	Yes
Hizmetli et al. (2005) [88]	SF and plasma	41 RA, 25H	No significant difference between RA patients and healthy controls	No
Oner et al. (2015) [89]	Serum	106 RA, 52H	No significant difference between RA patients and healthy controls	No
Otero et al. (2006) [100]	Plasma	31 RA, 18H	Markedly increased in RA patients	Yes
Allam et al. (2012) [101]	Serum	37 RA, 34H	Higher in RA patients	Yes
Anders et al. (1999) [91]	Serum	58 RA, 16H	No significant difference between RA patients and healthy controls	No
Popa et al. (2005) [90]	Plasma	31 RA, 18H	No significant difference between RA patients and healthy controls	No
Wislowska, M. et al. (2007) [97]	Serum	30 RA, 30OA	No difference between OA and RA	No
Chihara et al. (2020) [12]	Serum	136 RA, 78H	Higher in RA patients	No
Toussirot et al. (2013) [102]	Serum	30 RA, 51H	No difference between RA patients and healthy controls	No

SF, synovial fluid; H, healthy controls; RA, rheumatoid arthritis.

### 5.2. Leptin in Animal Models of RA

Many studies reported the strong pro-inflammatory role of leptin in mouse RA models [103]. In antigen-induced arthritis, a model of immune-mediated joint inflammation, leptin contributes to joint inflammation by modulating both humoral and cellular immunity. Synovial inflammation was diminished (low score of synovial thickness) in *ob/ob* mice as were TNFα, IL-1β, and IFN-γ, while IL-10 production was increased indicating an activation of the Th2 response [104]. In contrast, in zymosan-induced arthritis (ZIA), a model of proliferative arthritis, the resolution of joints inflammation was delayed in *ob/ob* and *db/db* mice and the severity of articular damage, as indicated by the histopathological scores, was higher in leptin-deficient mice compared to controls [105].

**Figure 2:**
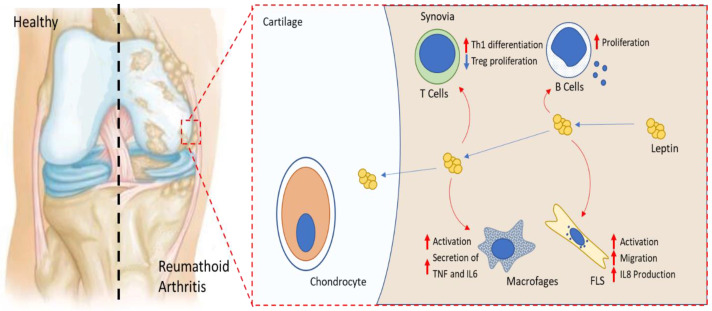
Leptin effect on RA key effector cells. Leptin enhances Th17 proliferation, B cell activation, and macrophage production of TNF and Il-6. It activates FLS migration and secretion of IL-8. It acts on chondrocytes by activating NOS expression and adhesion molecules leading to lymphocyte infiltration to the inflamed joints and degradation of articular cartilage.

Leptin increased the severity of K/BxN arthritis in mice, while administration of leptin receptor antagonist (Allo-aca) attenuated the disease severity. In a mild adjuvant-induced arthritis mouse model, leptin and leptin receptor antagonists reduced the number of arthritic joints and ameliorated the disease [106]. In another model of arthritis induced by collagen (CIA) in high-fat-diet-induced obese mice, mice developed peripheral leptin resistance and exhibited decreased disease severity and collagen-induced inflammation [107]. Leptin enhanced Th17 cell generation in splenic-cultured CD4+ T cells, while cultured naive T cells from *db/db* mice treated with leptin did not show any effect on Th17 differentiation indicating that this action is mediated via its receptor signaling (Figure 2). The same authors showed a significant elevation of leptin levels in synovium fluid of mice during CIA development, which was positively correlated to Th17 cells and IL-17 levels. Leptin intraarticular injection into CIA mice increased Th17 cells in joint tissue and exacerbated arthritis symptoms and synovial hyperplasia leading to enhanced cartilage degradation and bone erosion [108]. In septic arthritis induced by *Staphylococcus aureus*, leptin levels were decreased and its administration led to a decrease of disease severity and inflammatory response as evidenced by decreased IL-6 levels [109].

### 5.3. Leptin and Major Effector Cells in RA

Fibroblasts-like synoviocytes (FLS) are one of the potent cells participating in RA. They are the key effectors in synovium hyperplasia and are involved in bone destruction, local cytokine production, and enzymes degrading ECM synthesis [110]. Leptin induced RA FLS migration and angiogenesis by increased reactive oxygen species (ROS) production and antagonists of TNF, IL-6, and IL-1 attenuated leptin-induced ROS generation and FLS migration [111]. Additionally, Tong et al., indicated that leptin receptors, Ob-Rb and Ob-Rs, are expressed in synovial fibroblasts. IL-8 production was increased in synovial fibroblasts after leptin treatment for 24 h. This involved JAK2/STAT3, IRS-1/PI3K, Akt, and NF-κB pathways and the recruitment of p300, promoting, therefore, the inflammatory process which may play a key role in RA [69].

Leptin has been described to modulate bone homeostasis by both locally and centrally mediated mechanisms. It inhibits osteoclast differentiation in peripheral blood mononuclear cells (PBMCs) and murine spleen cells in bone culture via the RANKL/RANK/OPG system and thus contributes to the inhibition of bone resorption [112]. Leptin showed a direct effect synergistically with INF-gamma and also with IL-1 on chondrocytes. It induced NOS2 activation in murine and human primary chondrocytes via signaling pathways including JAK2, PI3k, Mek1, and p38 [63,113]. Moreover, it enhanced VCAM1 expression in human and murine chondrocytes through JAK2 and PI3K pathways, suggesting that leptin exacerbates cartilage degradation by activating factors involved in lymphocyte adhesion and leucocyte infiltration into the sites of inflamed joints [114].

The role of leptin in RA is not only associated with articular tissues, it might also have a potent effect on cell-mediated immune function. Accordingly, starvation of RA patients, which is correlated with decreased leptin levels, led to a reduction in CD4+ and CD8+ and a significant elevation of IL-4 production, proven to inhibit production of the pro-inflammatory cytokines in ex-vivo RA synovitis [115]. Furthermore, Lord et al. showed that exogenous leptin administration completely reversed the immunosuppressive effect induced by starvation in C57BL/6 mice. The same authors reported that leptin enhanced T cell proliferation by binding to its long isoform, Ob-Rb, expressed by lymphocytes. It differentially modulated the proliferation of naive (CD45RA^+^) and memory (CD45RO^+^) T cells and favored the T-cell response to pro-inflammatory phenotype by enhancing Th1 cytokine production and inhibiting Th2 response. Leptin also stimulated the expression of adhesion molecules ICAM-1 and VLA-2 on CD4+ T cells [116]. In addition, leptin promoted Th17 differentiation from naive T cells [108]. Th17 cells have been reported to be involved in the progression of arthritis and the pathogenesis of other autoimmune diseases [117].

RA is also associated with regulatory CD4^+^Foxp3^+^ T cells (Treg) defect [118] (Figure 2). Leptin receptor is greatly expressed on the Treg cell surface. Leptin neutralization by leptin monoclonal antibody (mAb) resulted in the reversion of the hyporesponsivness to anti-CD3 and anti-CD28 stimulation and promotion of Treg cell expansion. This proliferation was reversed by the addition of leptin recombinant. In vivo, leptin-deficient *ob/ob* mice showed higher amounts of circulating Treg cells [119]. Taken together, these results suggest that leptin might act as a negative signal for the expansion of Foxp3^+^CD25^+^CD4^+^ and might be a potential therapeutic approach for autoimmune disease. Leptin has also been described to regulate humoral immunity response. IgG, IgM, and IgA decreased in arthritic *ob/ob* mice and Ob-Rb mRNA was expressed in isolated B cells showing that leptin has a direct effect on these cells [104].

## 6. Leptin as a Potential Therapy in OA and RA

Current treatments of OA and RA are mainly focused on alleviating pain and do not completely fulfill the need of the patients. OA treatment based mainly on non-steroidal anti-inflammatory drugs (NSAIDs), corticosteroids, supplements including glucosamine and chondroitin, and inhibitors of matrix metalloproteinases have not shown relevant efficacy or the benefits are outweighed by side effects [120]. In RA, DMARDs showed a beneficial effect in ameliorating radiography damage but they were not able to reverse the disease [81]. Leptin at the crossroad between inflammation, metabolism, and immunity in joint diseases, could provide new therapeutic approaches to improving outcomes for patients suffering from rheumatic diseases and autoimmune disorders. Leptin activates the mTOR pathway, a key regulator of cartilage homeostasis, and induces cell senescence in chondrocytes [66]. It has been demonstrated that cartilage-specific deletion of mTOR in mice led to an increase in autophagy marker expression, and a decrease in chondrocyte apoptosis and expression of the catabolic factor MMP13. In human OA chondrocytes, rapamycin treatment resulted in suppression of S6K, and an increase in autophagy genes and anabolic factors aggrecan and type II collagen. It also reduced MMP13 and levels of the chemokines CCL5/RANTES and CCL2/MCP-1, suggesting that mTOR may be responsible for the regulation of imbalance between anabolic and catabolic processes in cartilage [121]. Hence leptin, as an activator of the mTOR/autophagy pathway, may be a therapeutic target for chondrocyte senescence and progression of cartilage degeneration. Leptin as a cross-link between obesity and OA, could offer new insights into the understanding of the mechanism by which weight loss improves OA symptoms [122]. Furthermore, adipose tissue is the main source of leptin and other adipokines causing inflammatory states mainly in obese people, thus, reducing fat mass and increasing physical exercises would improve obesity-related OA. Accordingly, acute starvation of RA patients, associated with reduced leptin levels, ameliorated the clinical symptoms of the disease including CRP level decrease and CD4+ T-cell activation [115], nonetheless, increased circulating leptin levels and leptin resistance are observed especially in obese patients. Thus, antagonizing leptin actions would be a possible therapeutic approach for RA. Accordingly, leptin mutants may be considered as antagonists and could be a hope for the control of leptin effects in the pathogenesis of autoimmune diseases. S120A/T121A binding site III mutant was found to have an affinity to CRH2 but unable to activate leptin receptor. It acts as a potent leptin antagonist and inhibits the activation of LepR in a dose-dependent manner [123]. Additionally, monoclonal human leptin antibodies against leptin or LepR could be a promising strategy. As described above, neutralizing leptin by a monoclonal antibody (mAb) promoted Treg proliferation. Furthermore, 9F8 attenuated leptin signaling as demonstrated by its inhibition of leptin-induced TNF-α expression and T-cell proliferation. Nevertheless, these antibodies should not affect the central actions of leptin and consequently lead to increased obesity and hyperphagia. Recombinant leptin was used to treat genetically deficient leptin patients [124]. Actually, there is a recombinant form of leptin promoted as a drug, known as recombinant methionyl human leptin (metreleptin). It was approved as a treatment for congenital or acquired generalized lipodystrophy. It has various metabolic effects on body weight, food intake, immunity, and brain function [125], however, no clinical assays were performed on its effect in cartilage degeneration. Therefore, it would be interesting to test metreleptin in joint-related diseases. Other alternatives targeting leptin signaling could also be developed. Accordingly, based on the previous studies supporting SOCS-3 as central to leptin resistance and its role in modulation of the leptin response in cartilage by regulating pro-inflammatory and catabolic effects [126], SOCS-3 could be a therapeutic target for the control of leptin actions and the prevention or treatment of OA especially in obese patients. In addition, enhancement of SHP2, a potent modulator of leptin signaling and functions in hypothalamus, could be an treatment approach for obese patients with leptin resistance [127].

## 7. Conclusions

The extensive research on leptin and its roles in RA and OA summarized in this review, indicate an advanced understanding of the role of this protein and its contribution to the cross-talking networks implicated in the pathogenesis of inflammation and immune-associated diseases. Its prevailing ability to promote cartilage metabolism and systemic and local inflammatory responses, not only in joints, but also in other immune cells and tissues, is further evidence of its key involvement in RA and OA development and progression. Notwithstanding, certain controversial results of published clinical studies could be attributed to the heterogeneity of the clinical confounders of the different study populations. Thus, well-designed, prospective, and multi-center research studies with large cohorts of patients may be warranted or, alternatively, big meta analyses might be proposed as a good potential answer to current questionable results.

Thus, leptin is emerging as a potential therapeutic target for the struggle of the degradative process observed in rheumatic patients. Several attractive approaches such as high-affinity leptin-binding molecules or administration of LepR antagonists are already underway. Despite that, many aspects of the concrete mechanisms of action of leptin in the development of such complex diseases as RA and OA are still not completely clear. Therefore, targeting these mechanisms without affecting the normal physiological role of leptin and other adipokines should be considered as well as pursuing the research of the unknown aspects of this molecule which remains a challenge for the future.
